# The transdiagnostic structure of mental effort avoidance

**DOI:** 10.1038/s41598-018-37802-1

**Published:** 2019-02-08

**Authors:** Edward H. Patzelt, Wouter Kool, Alexander J. Millner, Samuel J. Gershman

**Affiliations:** 1 Department of Psychology, Harvard University, Cambridge, USA; 2 Center for Brain Science, Harvard University, Cambridge, USA

## Abstract

The *law of least mental effort* states that, everything else being equal, the brain tries to minimize mental effort expenditure during task performance by avoiding decisions that require greater cognitive demands. Prior studies have shown associations between disruptions in effort expenditure and specific psychiatric illnesses (e.g., schizophrenia and depression) or clinically-related symptoms and traits (e.g., anhedonia and apathy), yet no research has explored this issue transdiagnostically. Specifically, this research has largely focused on a single diagnostic category, symptom, or trait. However, abnormalities in effort expression could be related to several different psychiatrically-relevant constructs that cut across diagnostic boundaries. Therefore, we examined the relationship between avoidance of mental effort and a diverse set of clinically-related symptoms and traits, and transdiagnostic latent factors in a large sample (*n* = 811). Only lack of perseverance, a dimension of impulsiveness, was associated with increased avoidance of mental effort. In contrast, several constructs were associated with less mental effort avoidance, including positive urgency, distress intolerance, obsessive-compulsive symptoms, disordered eating, and a factor consisting of compulsive behavior and intrusive thoughts. These findings demonstrate that deviations from normative effort expenditure are associated with a number of constructs that are common to several forms of psychiatric illness.

## Introduction

Each day we are faced with tasks that require mental effort. The level of effort depends on the difficulty of achieving the desired goal. Given that mental effort is a critical part of daily life, understanding the role of mental effort in psychopathology may provide key insights into psychiatric conditions that show deviations from exerting effort towards adaptive behavior. For example, drug seeking or compulsivity can be thought of as exercising effort towards the wrong goal. For individuals who become addicted to drugs, effort manifests as motivated planning to offset cravings; and similarly, counting in OCD is aimed at immediately reducing feelings of distress. Meanwhile, symptoms such as anhedonia and avolition can be thought of as a failure of effort exertion. Elevations in apathy are associated with a lack of interest, and likewise, avolition is a deficit in the initiation of self-directed and purposeful activities.

A number of studies have demonstrated that decision making that requires mental effort is disrupted in clinical populations with diagnoses of schizophrenia and depression (for a review, see Culbreth, Moran, & Barch, 2017) and in symptoms or personality traits such as anhedonia^1,2^ and apathy (for a recent review, see Le Heron, Apps, & Husain^3^). It has been argued these disruptions are due to abnormalities in the processes underlying the expenditure of effort^4,5^ (e.g., cognitive control, cost-benefit evaluations, and/or reward responsivity). Moreover, irregularities in these processes are putatively associated with a range of clinical diagnoses. However, these prior studies have focused on examining associations between effort-based decision making and specific clinically-related constructs or diagnoses, tying disrupted effort onto a single measure or diagnostic group. The limitation of this approach is that effort disruptions are not localized to a single diagnosis or construct, but are often associated with several symptoms and traits that cut across diagnostic boundaries.

We overcome the limitations of examining effort expenditure in a single psychological construct or diagnostic group by taking a dimensional approach to psychopathology in a large sample^6–8^. We measure several symptoms and traits, and obtain transdiagnostic latent factors to investigate associations between a well-defined measure of mental effort^9^, and several psychiatric constructs that transverse psychopathology.

Among the multiple ways to examine mental effort, one approach is to focus on people’s tendency to minimize effort expenditure (*law of least mental effort*). This idea is borrowed from a fundamental principle underlying most theories of *physical* effort, that people seek to minimize physical demands^10^. Preliminary evidence for the law of least mental effort comes from Tversky & Kahneman’s research^11^, which suggests that people use heuristics and other strategies to minimize cognitive demands. Recently, Kool and colleagues^9^ provided the first direct evidence for the principle of mental effort minimization in a novel paradigm called the demand selection task (DST). In this task, participants freely choose between two options that demand differing levels of mental effort. The high-demand option requires increased mental effort in the form of frequent task switching^12^, whereas the low-demand option necessitates less frequent task switching. Across many replications of this task, participants tend to prefer the less demanding course of action^9,13,14^. This avoidance of cognitive demand is consistent with the *law of least mental effort*^9,15^.

The DST differs from other effort paradigms in several key ways. First, in both the EEfRT^1^ (Effort Expenditure for Rewards Task) and COGED^16^ (Cognitive Effort Discounting) tasks the level of effort required for each choice is explicitly stated. In contrast, the DST requires participants to learn the difference in effort required between the low- and high-demand options, and therefore individual variability in effort expression may reflect variability in the learning process. Second, a number of effort paradigms^1,16–19^ provide rewards for greater effort exertion, whereas the DST does not provide rewards. Therefore, the DST may represent an individual’s preferred level of effort independent of a reward structure. Finally, the DST is specific to mental effort, whereas other paradigms measure physical effort with repeated finger presses^1,18^ or grip strength^19^. The differences between the DST and other effort tasks, and the lack of transdiagnostic studies, provides a unique opportunity to take a transdiagnostic approach to the avoidance of mental effort.

In the current study, we examine the expression of mental effort across the psychopathology spectrum. In addition to measuring a broad range of symptoms and traits, we produce three transdiagnostic factors (anxious depression, compulsive behavior and intrusive thought, and social withdrawal) that have been previously shown to be relevant to psychopathology and goal-directed behaviors^20,21^. Combining the DST with these psychiatric factors and several psychiatrically-relevant constructs will allow us to investigate the *law of least mental effort* transdiagnostically. We hypothesize that several constructs will be associated with greater avoidance of mental effort because theoretical models suggest common sources of dysfunction underlying the expression of effort in psychiatric illness^22,23^. If we find the expression of mental effort is associated with transdiagnostic constructs, it would suggest a common source of dysfunction in the processes underlying effort expression across several clinical diagnoses.

## Methods

### Participants

A total of 905 participants were recruited using Amazon Mechanical Turk. The participants provided informed consent, and the study and experimental protocols followed ethical guidelines and were approved by the Committee on the Use of Human Subjects (CUHS), the Institutional Review Board (IRB) for Harvard University. Participants were required to have U.S. residency, 90% approval rating, and 100 completed Mechanical Turk Human Intelligence Tasks. The study was completed over two sessions, and included the DST^9^, an adaptive IQ test^20,24^, nineteen self-report measures, and a sequential decision making task^21,25^. Participants were compensated $20. Exclusions included current drug and/or alcohol intoxication (n = 3) and demonstrated non-adherence on the IQ test (n = 72) and/or DST (n = 19). The IQ test questions became increasingly easier with incorrect responses, therefore participants with decreasing IQ scores indicated non-adherence or random responding. Participants endorsing mental health diagnoses were not excluded at a higher rate than those without (see Supplemental). The final sample included 811 participants that were 47.9% female and 52.1% male, ranged in age from 18–73 years old (M = 34.9, SD = 10.2) and had an average IQ of 99.1 (SD = 9.7). Participants were 78.4% Caucasian, 8.8% Black/African American, 6.2% Hispanic/Latino, 6.0% Asian/Asian American, and 0.5% Native American/Other. Table 1 contains self-reported clinical diagnostic and treatment information.

**Table 1:** Self-reported demographics.

Self-reported Clinical Characteristics		n = 811
Endorsed Specific Diagnosis	37.24%	(302)
2 Diagnoses	13.56%	(110)
>=3 Diagnoses	6.78%	(55)
*Past Treatment*: Any Treatment	31.32%	(254)
Partial, Inpatient, Residential	8.38%	(68)
*Current Treatment:* Any Treatment	20.47%	(166)
Partial, Inpatient, Residential	4.43%	(36)
Psychiatric Medication	9.49%	(77)

### Self-report measures

Table 2 provides definitions and sources for nineteen psychiatric constructs from self-report measures completed by the participants. In a separate but related project on sequential decision making and psychopathology^21^, we replicated and extended the findings of Gillan and colleagues^20^ using overlapping self-report measures. Like these former studies, nine of the self-report measures in the current study can be summarized with three latent factors (anxious depression, compulsive behavior and intrusive thought, and social withdrawal). We generated the scores for these factors using the factor loadings from Gillan and colleagues^20^. The measures included in these factors are: Apathy Evaluation Scale^26^, trait portion of the State-Trait Anxiety Inventory^27^, Alcohol Use Disorders Identification Test^28^, Barratt Impulsiveness Scale-11^29^, Zung Depression Scale^30^, Eating Attitudes Test^31^, Obsessive-Compulsive Inventory-Revised^32^, Short Schizotypy Scale^33^, and Social Anxiety Scale^34^.

**Table 2:** Descriptions of Psychiatric Constructs.

Construct	Description
Perseverance (lack of)	Difficulty maintaining attention and vulnerability to intrusive and interfering information^42^
Rumination	Thinking repetitively and passively about negative emotions, with a focus on distress^37^
Sensation Seeking	Willingness to take risks (financial, legal, physical) for novel-intense experiences^42^
Premeditation (lack of)	Difficulty considering the long-term consequences of actions^42^
Barratt Impulsiveness^a^	Dimensions of impulsivity (attentional, motor, non-planning impulsiveness)^29^
Social Anxiety^a^	Anxiety and avoidance of social situations likely to induce fear of evaluation^34^
Alcohol Use^a^	Hazardous and harmful alcohol consumption, drinking behavior, and alcohol related problems^28^
Trait Anxiety^a^	Stable tendency to experience and attend to negative emotions^27^
Anxiety Sensitivity	Tendency to respond fearfully to physiological cues of anxiety (e.g. increased heart rate)^36^
Apathy^a^	Lack of motivation not due to diminished consciousness, cognitive impairment, or emotional distress^26^
Schizotypy^a^	Unusual experiences, cognitive disorganization, introvertive anhedonia, and impulsive non-conformity^33^
Negative Urgency	Strong immediate need to avoid negative emotions or physical sensations^42^
Uncertainty Intolerance	Tendency to consider possibility of negative event unacceptable, regardless of likelihood^35^
Depression^a^	Affective (e.g. sad), physiological (e.g. sleep disturbance), and psychological (e.g. hopeless) symptoms^30^
Emotion Dysregulation	Difficulty with emotional awareness, understanding, acceptance, regulation, and perseverance^38^
Disordered Eating^a^	Dieting, bulimia and food preoccupation, and oral control^31^
Obsessive-Compulsive^a^	OCD symptoms (checking, washing, obsessing, mental neutralizing ordering, hoarding, doubting)^32^
Distress Intolerance	Reduced capacity to withstand and experience negative psychological states^39^
Positive Urgency	Tendency to act rashly or maladaptively in response to positive mood states^41^

Self-report measures with exact (or abbreviated) definition from source article. Organized according to ascending coefficient mean (see Fig. 3). ^a^Scale included in Gillan *et al*.^20^ and used to derive psychiatric factors (anxious depression, compulsive behavior and intrusive thought, and social withdrawal).

We measured ten additional constructs because they encompass personality traits (i.e., intolerance of uncertainty, rumination, emotion dysregulation, distress intolerance, impulsivity) that underlie prominent theories of psychiatric illness. These measures were, the Intolerance of Uncertainty Scale^35^, Anxiety Sensitivity Index-3^36^, Ruminative Response Scale^37^, Difficulties with Emotion Regulation Scale^38^, Distress Tolerance Scale^39^, and the UPPS-P Impulsivity Scale^40–42^ (the UPPS-P is comprised of positive & negative urgency, sensation seeking, lack of premeditation, and lack of perseverance). We did not conduct a factor analysis with these measures because the sample size is underpowered and we sought to stay consistent with Patzelt and colleagues^21^.

### Demand selection task

**Figure 1:**
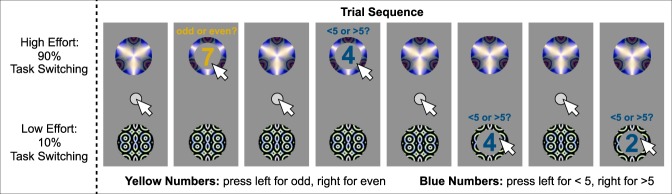
Demand selection task. The participant is presented with the choice between two abstract patches representing the cues. A probe appears as a colored number following cue selection, and the participant responds with a parity judgement on yellow numbers (e.g., odd or even), or magnitude judgement on blue numbers (e.g., <5 or >5). Note, the magnitude and parity rules are displayed above, but not visible to the participant during the task. The top cue is the *high demand* option and requires frequent task switching between parity and magnitude judgements (i.e., 90% of the time; high mental effort). The bottom cue is the *low demand* option and requires infrequent task switching between parity and magnitude judgements (i.e., 10% of the time; low mental effort). Participants completed 300 trials across 4 blocks and 4 different cue pairs.

The study used an abridged version of the DST first reported in Experiment 3 of Kool and colleagues^9^ and also used in Experiment 1 of Gold and colleagues (2014), and it was adopted for use on Mechanical Turk (https://github.com/wkool/demandavoidance). In this paradigm, the participant is presented with two abstract color patches representing the cues (Fig. 1). The participant chooses a cue, and a probe appears as a blue or yellow number between 1 and 9. Participants were instructed to make a parity judgment on yellow numbers (i.e., odd or even) or a magnitude judgement on blue numbers (i.e., >5 or <5).

One cue represented the *high-demand* option, whereas the other represented the *low-demand* option. When choosing the high-demand option over several trials, the participant would be required to switch tasks between parity and magnitude judgments 90% of the time. Alternatively, when choosing the low-demand option consistently over several trials, the participant would be required to switch tasks between parity and magnitude judgements 10% of the time. Thus, the high-demand option required more task switching relative to the low-demand option, and therefore greater cognitive effort^9,12,43^.

There were three rounds of training where participants first practiced 10 trials making parity or magnitude judgements with accuracy feedback on every trial. Next, participants completed 10 trials with feedback at the end, to ensure the correct response mappings. Finally, participants practiced 20 trials of the full task by choosing a cue and responding with a parity or magnitude judgment depending on the color of the displayed number. The participants completed 300 trials of the DST with 75 trials across 4 blocks and 4 cue pairs. The location and appearance of the cues remained fixed within block while varying across block.

### Analyses

#### Measuring mental effort

Following prior work^9^, we measured the degree of demand avoidance as the proportion of trials on which participants chose the low demand cue. Accuracy was calculated separately for low demand and high demand trials. Participants were excluded (N = 19) if their overall task performance reflected random responding, which was calculated using a binomial distribution to determine the minimum number of correct responses (i.e., 165/300 trials) needed to surpass the 50% chance accuracy threshold.

#### Demand avoidance bias and psychiatric constructs (Bayesian regression)

Bayesian regression was used to estimate the relationship between the psychiatric constructs and demand avoidance^44^. This differs from frequentist approaches to linear analysis in several ways. Frequentist linear regression produces a point estimate of the regression coefficients. This is often reported in addition to 95% confidence intervals, which indicate that over many repetitions of the study, 95% of intervals will contain the population value. As noted by Baldwin & Larson^45^, confidence intervals are often misinterpreted as the interval that contains probable values of the beta coefficient. However, in frequentist theory, the confidence interval indicates confidence in the method (i.e., the study design) that produced the beta estimate. This point estimate is subjected to significance testing for rejection of the null hypothesis. The frequentist interpretation is binary: either there is a lack of a relationship between the predictor and the dependent variable (i.e., the null hypothesis is true), or the null hypothesis is rejected.

In contrast, Bayesian linear regression incorporates probability into the estimate of beta values for the regression coefficient. A posterior probability distribution over possible beta values is produced that can be summarized using credible intervals, indicating the concentration of betas around the mean or median. This allows us to quantify the magnitude of the effect between the psychiatric constructs and demand avoidance because we can estimate the probability that a positive or negative relationship exists (rather than accepting or rejecting the null). We report the 95% highest posterior density (HPD) interval. This indicates the range of coefficient values around the posterior mean (β_mean_) that contains 95% of the posterior probability density. The percentage of the posterior density that exceeds or falls below 0 indicates the probability of a positive relationship (i.e., construct is associated with more demand avoidance), or negative relationship (i.e., construct is associated with less demand avoidance). We used the brms package^46^ with the default prior σ ~ *student-t*(3,0,10) and separately regressed each self-report measure onto the proportion of low demand choices (i.e., demand avoidance) while controlling for age, IQ, and gender.

We also accounted for collinearity (correlations reported in supplemental information, Fig. S3) by entering all the self-report measures into a single regression with elastic net regularization using the caret^47^ and glmnet^48^ packages in R. The analysis used 20-fold cross-validation with 10 repeats to establish the best alpha and lambda parameters for the elastic net.

### Transdiagnostic factors

We derived psychiatric factors by regressing the published factor loadings from Gillan *et al*.^20^ onto the item level scores for nine self-report measures that overlapped with their study (see Table 2). This generated individual factor scores for the participants in our study corresponding to the three psychiatric factors: anxious depression, compulsive behavior and intrusive thought, and social withdrawal. We regressed all three measures concurrently onto the proportion of low demand choices, again controlling for age, IQ, and gender.

To confirm the factor structure produced by Gillan and colleagues we performed a confirmatory factor analysis (CFA) with the lavaan package^49^. The latent variable definitions (e.g., anxious-depression) in the specified model describe how the factors manifest from the observed self-report items. We specified the factor structure according to the latent factor model provided by Gillan and colleagues and the fit was measured by two metrics that account for model complexity, the RMSEA and SRMR^50^. We then regressed the CFA loadings onto the proportion of low demand choices to establish if loadings generated solely from our data would produce similar results to the Gillan *et al*.^20^ loadings.

## Results

### Demand selection task

**Figure 2:**
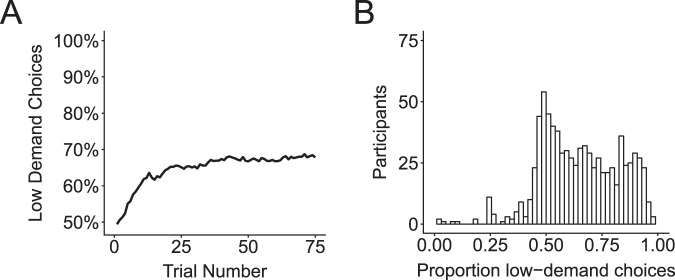
(**A**) Participants displayed a bias towards minimizing cognitive demand by more frequently selecting the *low demand* cue that required task switching between parity and magnitude judgments 10% of the time. (**B**) Distribution of the proportion low-demand choices across participants.

On average, participants selected the low-demand option 65% of the time (SD = 0.18; Fig. 2). A one-sample t-test showed participants significantly preferred the low-demand option over the high demand option (t(810) = 23.59, p < 0.001). Mean accuracy for the low-demand option was 0.95 (SD = 0.063) and for the high-demand option was 0.93 (SD = 0.073), and these differed significantly from each other (t(1587.3) = 5.51, p < 0.001). IQ was significantly positively correlated with demand avoidance (r(792) = 0.21, p < 0.001), indicating increasing IQ was associated with a bias towards greater demand avoidance (i.e., less mental effort). Age was uncorrelated with demand avoidance (r(792) = −0.01, p = 0.71).

### Demand avoidance and self-report measures

**Figure 3:**
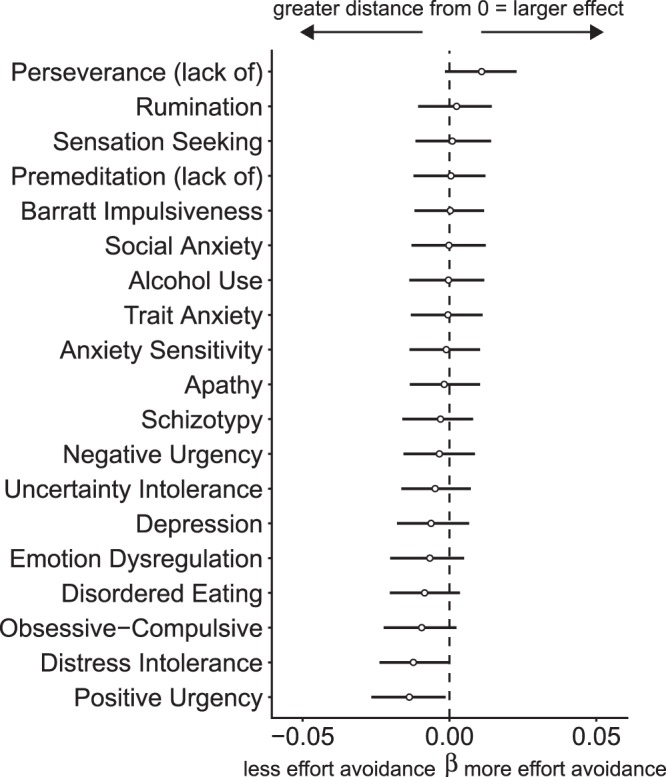
Coefficient plot where the beta indicates the estimated slope of the line relating severity of the self-report measures and proportion of low demand choices while controlling for age, IQ, and gender. Credible intervals are displayed containing 95% of the posterior probability density around the mean, organized according to ascending coefficient mean. Negative beta intervals are evidence for less demand avoidance (more mental effort) and positive beta intervals are evidence for more demand avoidance (less mental effort).

Figure 3 shows the results of separate regressions for each self-report measure. The distance from zero of the mean beta coefficients and credible intervals indicate the magnitude of the relationship between demand avoidance and the self-report measures. Table 3 reports the posterior probability of beta less than or greater than zero. Almost the entire posterior distribution of possible betas for lack of perseverance was placed on values greater than zero (96.0% positive), indicating that the self-report measure was associated with a greater demand avoidance bias (i.e., less mental effort). Alternatively, positive urgency (1.9% positive) and distress intolerance (2.1% positive) had posterior data distributions where at least 95% of the beta estimates were negative. The obsessive-compulsive symptoms (6.2% positive), and disordered eating (8.5% positive) had posterior beta distributions where at least 90% of the beta estimates were negative, which is below the 95% HPD, yet still indicates high certainty about the association between these measures and mental effort. Thus, increases in these constructs were associated with less demand avoidance bias, meaning people high in these constructs exerted relatively *more* mental effort than individuals with low scores on these scales. Additionally, several self-report measures were associated with a lesser demand avoidance bias but there was high uncertainty in these relationships (i.e., less than 90% but more than 50% of the posterior was negative). The regression using elastic net largely replicated the same pattern of results with parameter values of alpha = 0.55 and lambda = 0.008 (Supplemental Fig. S4). There was no difference in demand avoidance as a function of the presence of a diagnosis as shown in the Supplemental Information.

**Table 3:** Posterior probabilities of relationship between self-report meaures and demand avoidance.

*Construct*	Neg (<0)	Pos (>0)
Perseverance (lack of)	4.0%	96.0%
Rumination	35.1%	64.9%
Sensation Seeking	44.0%	56.0%
Premeditation (lack of)	46.2%	53.8%
Barratt Impulsiveness	47.8%	52.2%
Social Anxiety	51.2%	48.8%
Alcohol Use	51.7%	48.3%
Trait Anxiety	52.3%	47.7%
Anxiety Sensitivity	57.1%	42.9%
Apathy	61.3%	38.7%
Schizotypy	69.0%	31.0%
Negative Urgency	71.3%	28.7%
Uncertainty Intolerance	78.0%	22.0%
Depression	84.7%	15.3%
Emotion Dysregulation	85.8%	14.2%
Disordered Eating	91.6%	8.5%
Obsessive-Compulsive	93.8%	6.2%
Distress Intolerance	98.0%	2.1%
Positive Urgency	98.1%	1.9%

Table showing the posterior probability density over β (the coefficient relating self-report measure severity to low demand choices) that each self-report measure is associated with decreases (i.e., negative <0; more mental effort) or increases (i.e. positive >0; less mental effort) in demand avoidance. The more the posterior probability density is <0 or >0, the higher the probability that there is a relationship between the measure and the expression of mental effort.

### Demand avoidance and psychiatric factors

**Figure 4:**
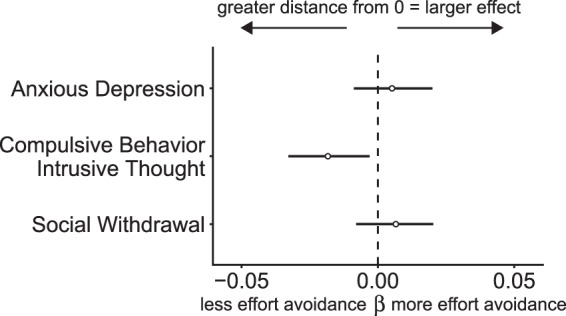
Coefficient plot where the beta indicates the estimated slope of the line relating severity of the transdiagnostic psychiatric factors and proportion of low demand choices while controlling for age, IQ, and gender. Credible intervals are displayed containing 95% of the posterior probability density around the mean. Negative beta intervals are evidence for less demand avoidance (more mental effort) and positive beta intervals are evidence for more demand avoidance (less mental effort). Factors derived from nine of the self-report measures indicated in Table 1.

Figure 4 shows the mean beta coefficients and credible intervals when regressing the three psychiatric factors onto demand avoidance concurrently, and Table 4 reports the corresponding posterior density of the possible beta values around 0. The compulsive behavior and intrusive thought factor was strongly associated with a reduced demand avoidance bias (0.9% positive) with most of the posterior distribution less than zero. In contrast, the anxious-depression and social withdrawal factors were associated with a greater demand avoidance bias, but there was greater uncertainty in these associations (anxious depression = 76.0% >0, social withdrawal = 82.2% >0).

**Table 4:** Posterior probabilities of relationship between psychiatric factors and demand avoidance.

*Construct*	Neg (<0)	Pos (>0)
Anxious-depression	24.0%	76.0%
Compulsive Behavior & Intrusive Thought	99.1%	0.9%
Social Withdrawal	17.8%	82.2%

Table showing the posterior probability density over β (the coefficient relating transdiagnostic factor severity to low demand choices) that each factor is associated with decreases (i.e., negative <0; more mental effort) or increases (i.e. positive >0; less mental effort) in demand avoidance. The more the posterior probability density is <0 or >0, the higher the probability that there is a relationship between the factor and the expression of mental effort.

We confirmed the factor structure reported by Gillan and colleagues using a CFA with two indices indicating good (RMSEA: 0.059), and acceptable (SRMR: 0.075) fits^50^. In addition, when the CFA loadings were regressed onto proportion low-demand choices the results were consistent with our results using the Gillan *et al*.^20^ loadings, demonstrating that the compulsive behavior and intrusive thought factor was associated with less effort avoidance (Supplemental Information Fig. S5).

## Discussion

The *law of least mental* effort states that, everything else being equal, we seek to minimize cognitive demands^9,15^. In this study, we investigated the relationship between mental effort and transdiagnostic measures of psychopathology in a large online sample. Former research has largely focused on a single diagnostic category or trait. In contrast, our study measured a broad range of symptoms, traits, and factors that cut across diagnostic boundaries. While increasing scores on lack of perseverance (a facet of impulsiveness), were associated with a greater avoidance of cognitive effort, increasing scores on several constructs were associated with less avoidance of cognitive effort. We found that (1) increasing lack of perseverance was associated with greater avoidance of cognitive effort; (2) increasing positive urgency, distress intolerance, obsessive-compulsive symptoms, and disordered eating were associated with less avoidance of cognitive effort (i.e., all had >90% of the posterior density distributions indicating avoidance decreases); and (3) increasing scores on a transdiagnostic psychiatric factor encompassing compulsive behavior and intrusive thought were also associated with less avoidance of cognitive effort. In addition, increasing scores on several of the self-report measures had more uncertain associations, but indicated less avoidance of cognitive effort (Fig. 3, Table 3).

Prior research on effort-based decision making has largely focused on a single phenomenon (e.g., anhedonia) or diagnostic category (e.g., schizophrenia). We used several transdiagnostic symptoms, traits, and factors to show specific associations between mental effort and various aspects of psychopathology. Lack of perseverance is defined as difficulty completing challenging or boring tasks^42^. Since this is a self-report measure of effort, this finding provides convergent validity with the DST’s behavioral index of effort avoidance. This is interesting because lack of perseverance has also been associated with several domains of psychopathology including depression, borderline personality traits, disordered eating, alcohol and substance use^51^. While lack of perseverance has been associated with an increased vulnerability for the development of schizophrenia^52^, other research indicates no association between lack of perseverance and a diagnosis of schizophrenia^53^. The current study does not provide evidence as to the relationship between lack of perseverance and DSM diagnoses such as major depressive disorder or schizophrenia. However, increased lack of perseverance may represent a transdiagnostic personality vulnerability within some forms of psychopathology. Further research is needed to establish the association between lack of perseverance and behavioral effort disruption commonly seen in clinical diagnoses.

With the exception of lack of perseverance, several scales were associated with less avoidance of cognitive effort, demonstrating individuals with higher scores on these constructs chose the high-effort option more frequently than those with lower scores. There are several possibilities to contextualize these findings.

First, our findings that increased psychopathology on several scales was associated with increased mental effort somewhat parallel one experiment in Gold and colleagues (2014); one of their experiments showed that patients with a diagnosis of schizophrenia chose the high effort option slightly more frequently. A forced choice condition in a control experiment (e.g., instructions to select the higher, or lower, effort cue) suggests that greater effort expenditure in the patient group may have arisen due to a deficit in adequately estimating the effort costs of the two response options. This is consistent with a cost-benefit account of mental effort^5,54,55^ and suggests that other forms of psychopathology may be associated with a disruption in the cost-benefit analysis underlying effort expression. However, Gold and colleagues (2014) were unable to demonstrate the main effect of demand avoidance, so it is difficult to determine the nature of the observed deficits in the schizophrenia group. In contrast, we were able to demonstrate the main effect of demand avoidance, so conclusions driven by comparison of the two studies are limited.

Second, Shenhav and colleagues^5^ propose that effort is comprised of a set of individual characteristics (e.g., information-processing capacity) and cognitive processes (e.g., performance monitoring) that mediate between task demands and task performance. These processes are disrupted in a range of psychiatric illnesses, and therefore another possibility is that the constructs associated with greater effort reflect a deficit in attention, performance monitoring, or other cognitive control abilities^56,57^. These individuals are not choosing the high effort option more because they prefer it, but because they fail to sustain the representation of the two cues simultaneously during the task. Our data cannot speak to this possibility and future research may benefit from combining effort tasks with other cognitive control paradigms to increase specificity when examining relationships between aspects of cognition and psychopathology.

A third possibility is that, individuals who are elevated on these clinically-related constructs are aware of which option requires lower effort and just prefer the high effort course of action. Evidence for this possibility could manifest as a scenario in which these participants show similar task accuracy as participants low on the clinically-related constructs and just choose to select the high effort option more frequently. Unfortunately, the current data cannot provide strong evidence for this possibility because accuracy and effort are confounded: the high effort option is more difficult, and therefore choosing this option more will decrease accuracy. In an exploratory analysis, we focused on subjects with 90% accuracy or above, and the results remained largely consistent (Supplemental Information). This supports the idea that participants were engaged with the task and deviations of effort were not simply due to random responding. Therefore, three possible mechanisms driving choosing the high effort option more frequently are (1) difficulty estimating the cost of the two response options^43^, (2) deficits in cognitive processing (e.g., cognitive control) that mediates between task demands and task performance^5^, and/or (3) a preference for greater mental effort.

Our results also diverge from a large literature indicating that psychopathology is generally associated with reductions of effort (for reviews see Culbreth *et al*.^4^; Pessiglione *et al*.^58^. However, in addition to the findings of Gold and colleagues indicating greater mental effort, there have been other ambiguities in this research. In a case-control study of major depressive disorder it was found that patients exerted less effort (relative to controls), yet within the patient group higher depressive symptoms were correlated with greater effort^59^. Using the same task (i.e., EEfRT), McCarthy and colleagues^60^ found a similar result in patients with a diagnosis of schizophrenia, who exhibited less overall effort, but demonstrated a positive association between effort exertion and negative symptom severity^60^. Meanwhile, another recent study using the EEfRT task found social anhedonia was associated with greater effort exertion^2^. These findings suggest that the relationship between the expression of effort and psychopathology may be more nuanced than anticipated by theories^4,58^ in which psychopathology is associated with effort reductions. Moreover, across studies supporting both reductions and increases in effort, the paradigms have incentivized participants to increase effort. For example, in the EEfRT task^1^, participants are provided more reward for more presses of the spacebar. As noted above, the DST does not provide greater reward for more effort expenditure. Therefore, the DST may comprise a different set of intervening processes that affect the expression of mental effort, due to the costs associated with action itself (rather than those due to the association between actions and outcomes).

The DST also differs from other effort-based decision-making tasks because participants in the DST are required to learn the effort required by each cue. Moreover, the DST only requires mental effort rather than the physical effort required in the COGED^16^, EEfRT^1^, and grip strength task^19^. It has been suggested that physical effort and mental effort may rely on the same underlying motivation system in the brain^61^, but behavioral performance may depend upon task-specific systems that differ between mental and physical effort. An open question is whether the effort deviations we observed were localized to this common motivational system, and to what extent the effort disruptions in the DST would generalize to other tasks. The addition of rewards to the DST could shift the observed results; however, it is also possible that we observed a trait-like deficit that would be consistent across other forms of effort-based decision making.

The compulsive behavior and intrusive thought factor demonstrated the same pattern of results as several self-report measures, showing a reduced avoidance of cognitive effort. The results for the anxious-depression and social withdrawal factors demonstrated a more uncertain pattern of results that was associated with greater avoidance of mental effort. These three factors were derived from nine scales that cut across diagnostic categories^20^. For example, the compulsive behavior and intrusive thought factor contained high loadings from items on the obsessive-compulsive inventory^32^ and the disordered eating questionnaire^31^. This suggests that this factor may be associated with cognitive deficits across several domains of psychopathology, perhaps reflecting general difficulties with cognitive control and/or estimating the costs associated with mental effort.

The main limitation of the current investigation was our inability to disentangle the association between psychiatric constructs and various processes underlying the expenditure of mental effort. As noted by others^5^, this limitation arises because the expenditure of effort is affected by multiple cognitive processes of the individual, and their relationship to psychopathology requires more systematic approaches (e.g., computational modeling) to concurrently examine, and formalize, the processes driving effort expression. Debriefing questions or forced choice trials may have aided in discriminating among the various underlying mechanisms driving effort disruptions such as preference for greater effort, difficulty estimating the effort cost of the two options, and/or cognitive processing deficits. Relatedly, learning the effort costs associated with each cue likely requires working memory processes that are disrupted in schizophrenia^62^, and we were unable to test for this possibility. In addition, we were unable to formally diagnose our sample with trained clinicians. The MTurk sample may not faithfully represent the general population, and specific disorders may have different relationships with mental effort. However, the relationships we report are based on widely used self-report measures that reflect common constructs in the study of psychopathology.

Another limitation is that the self-report measures are heterogenous in themselves, comprised of many different questions that may measure many different aspects of the proposed construct, further occluding the specificity the construct and its relationship with effort. Using the transdiagnostic factor approach we were able to partially overcome this challenge. However, we were unable to conduct an exploratory factor analysis on all the measure items concurrently due to the large number of items (385) and low subject-to-variable ratio (~2:1). Future research with a larger sample could identify additional factors and provide a more compact analysis by reducing the self-report measures into fewer dimensions. In addition, network^63^ and computational approaches to the structure of psychopathology^64^ will be needed to further parse dissociable aspects of psychiatric illness and look at relationships with cognition.

The current study demonstrated that various aspects of psychopathology are associated with deviations from the *law of least mental effort*. Our findings demonstrate the specificity that can be achieved with transdiagnostic approaches to the study of cognition. Choosing the low effort option more frequently is associated with a personality trait characterized by lack of perseverance. We report that choosing the high effort option more frequently is associated with elevations on several psychiatrically-relevant constructs, including positive urgency, distress intolerance, obsessive-compulsive symptoms, disordered eating, and a transdiagnostic psychiatric factor encompassing compulsive behavior and intrusive thought. This suggests that some psychopathological disruptions may be associated with increased effort expression, rather than reduced effort expression as suggested by common theoretical models of effort-based decision-making^4,58^. The current study has significant clinical implications, showing that symptoms, traits, and transdiagnostic dimensions that cut across diagnostic boundaries are associated with deviations in the expression of mental effort. Furthermore, these transdiagnostic abnormalities in effort exertion may correspond to challenges exerting the appropriate level of mental effort for the tasks of daily life.

## Supplementary information

Supplementary Information

## Data Availability

The datasets generated during and/or analyzed during the current study are available from the corresponding author upon request consistent with the IRB data sharing protocol.
