# Supplementary material for: The transdiagnostic structure of mental effort avoidance
Source: Sci Rep. 2019 Feb 8;9:1689. doi: 10.1038/s41598-018-37802-1 (PMC6368591; doi:10.1038/s41598-018-37802-1)
Supplement: Supplementary file 1 — Supplementary Information [file 41598_2018_37802_MOESM1_ESM.pdf]

Supplemental information:

The transdiagnostic structure of mental effort avoidance

Edward H. Patzelt[1,2*] MA, Wouter Kool[1] PhD, Alexander J. Millner[1] PhD, Samuel J. Gershman[1,2] PhD

[1]Department of Psychology, Harvard University

[2]Center for Brain Science, Harvard University

Supplemental tables: 4

Supplemental figures: 7

Supplemental tables and figures

Supplemental table 1. Posterior probabilities of relationship between self-report measures and demand avoidance (excluding < 90% accuracy; N = 723)

Construct	Neg (< 0)	Pos (> 0)
Perseverance (lack of)	2.4%	97.6%
Rumination	23.2%	76.8%
Anxiety Sensitivity	25.9%	74.1%
Apathy	37.8%	62.3%
Premeditation (lack of)	39.0%	61.0%
Barratt Impulsiveness	45.3%	54.7%
Social Anxiety	47.1%	52.9%
Trait Anxiety	50.2%	49.8%
Schizotypy	54.8%	45.2%
Sensation Seeking	60.4%	39.6%
Negative Urgency	65.6%	34.4%
Uncertainty Intolerance	67.9%	32.2%
Emotion Dysregulation	73.0%	27.0%
Alcohol Use	74.0%	26.0%
Depression	77.8%	22.2%
Disordered Eating	78.2%	21.8%
Distress Intolerance	94.5%	5.6%
Positive Urgency	94.9%	5.1%
Obsessive-Compulsive	97.8%	2.2%

Supplemental table 1. Participants with less than 90% accuracy were excluded. Table showing the posterior probability density over β (the coefficient relating self-report measure severity to low demand choices) that each self-report measure is associated with decreases (i.e., negative <0; more mental effort) or increases (i.e. positive > 0; less mental effort) in demand avoidance. The more the posterior probability density is <0 or >0 , the higher the probability that there is a relationship between the measure and the expression of mental effort.

Supplemental table 2. Posterior probabilities of relationship between psychiatric factors and demand avoidance (excluding < 90% accuracy; N = 723)

Construct	Neg (< 0)	Pos (> 0)
Anxious-depression	99.3%	0.7%
Compulsive Behavior & Intrusive Thought	18.2%	81.9%
Social Withdrawal	19.3%	80.8%

Supplemental table 2. Participants with less than 90% accuracy were excluded. Table showing the posterior probability density over β (the coefficient relating transdiagnostic factor severity to low demand choices) that each factor is associated with decreases (i.e., negative <0; more mental effort) or increases (i.e. positive > 0; less mental effort) in demand avoidance. The more the posterior probability density is <0 or >0 , the higher the probability that there is a relationship between the factor and the expression of mental effort.

Measure	Mean	SD	Min	Max
Age	34.93	10.18	18.00	73.00
IQ	99.13	9.73	81.02	126.82
Anxiety Sensitivity	17.29	13.60	0.00	68.00
Negative Urgency	24.02	7.90	12.00	48.00
Premeditation (lack of)	18.87	5.40	11.00	41.00
Perseverance (lack of)	18.27	5.80	10.00	39.00
Sensation Seeking	27.73	8.50	12.00	48.00
Positive Urgency	22.58	8.75	14.00	54.00
Distress Intolerance	40.60	13.71	19.00	71.00
Emotion Dysregulation	86.56	17.44	36.00	146.00
Uncertainty Intolerance	63.73	23.57	27.00	131.00
Rumination	42.78	13.71	22.00	84.00
Alcohol Use	3.85	4.48	0.00	34.00
Apathy	32.64	9.97	18.00	64.00
Barratt Impulsiveness	56.84	12.21	0.00	98.00
Obsessive-Compulsive	9.89	10.54	0.00	54.00
Depression	37.49	10.57	0.00	74.00
Disordered Eating	56.56	17.67	0.00	149.00
Schizotypy	12.50	5.97	1.00	32.00
Social Anxiety	43.85	16.68	0.00	96.00
Trait Anxiety	39.41	14.62	0.00	78.00
Anxious-Depression Factor	3.32	1.01	1.60	6.07
Compulsive-Intrusive Factor	2.35	0.94	0.65	5.59
Social Withdrawal Factor	2.84	1.07	1.38	5.77

Supplemental table 3. Means, standard deviation, and range of analysis variables.

	Excluded	Not Excluded	Total
**Diagnosis**	43	302	**345**
**No Diagnosis**	51	509	**560**
**Total**	**94**	**811**	**905**

χ^2 = 2.236, with 1df, p = 0.1348

Supplemental table 4. Participants with a diagnosis were not excluded at a higher rate than those without a diagnosis.

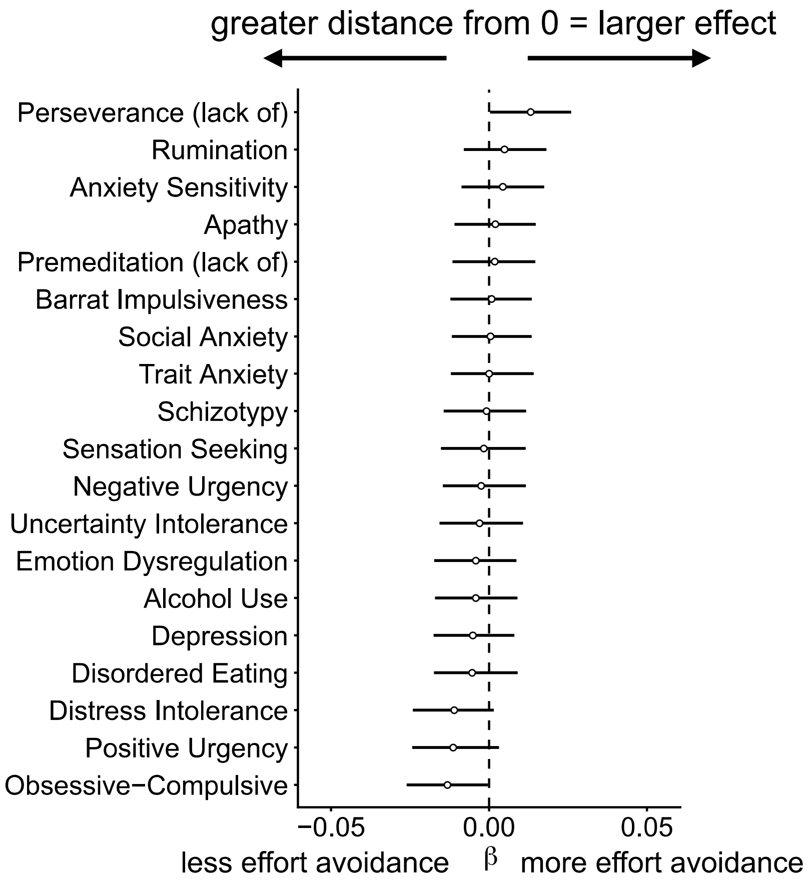

Supplemental figure 1. Participants with less than 90% accuracy were excluded. Coefficient plot where the beta indicates the estimated slope of the line relating severity of the self-report measures and proportion of low demand choices while controlling for age, IQ, and gender. Credible intervals are displayed containing 95% of the posterior probability density around the mean, organized according to ascending coefficient mean. Negative beta intervals are evidence for less demand avoidance (more mental effort) and positive beta intervals are evidence for more demand avoidance (less mental effort).

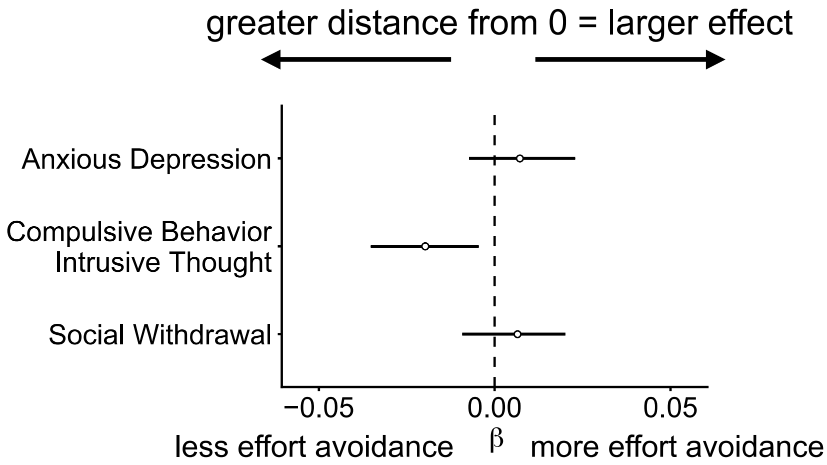

Supplemental figure 2. Participants with less than 90% accuracy were excluded. Coefficient plot where the beta indicates the estimated slope of the line relating severity of the transdiagnostic psychiatric factors and proportion of low demand choices while controlling for age, IQ, and gender. Credible intervals are displayed containing 95% of the posterior probability density around the mean. Negative beta intervals are evidence for less demand avoidance (more mental effort) and positive beta intervals are evidence for more demand avoidance (less mental effort). Factors derived from nine of the self-report measures indicated in table 1.

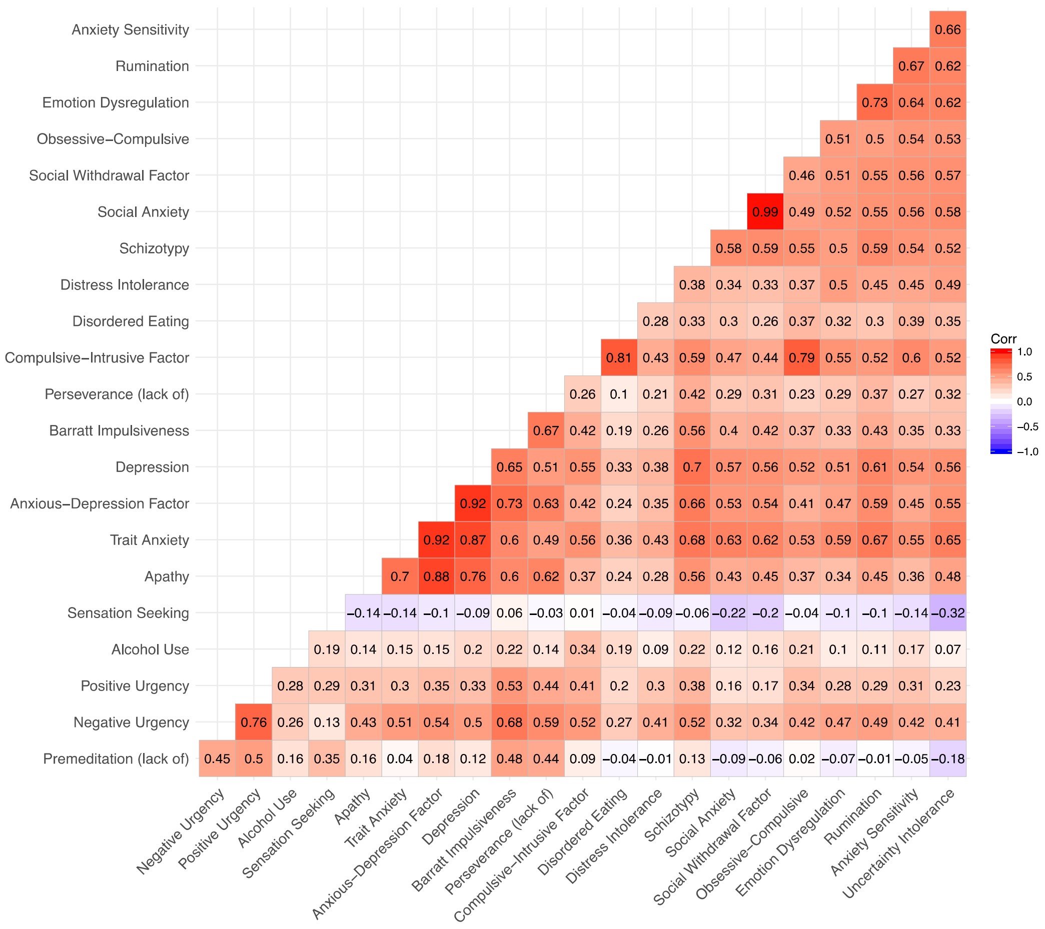

Supplemental figure S3. Correlations among the measures.

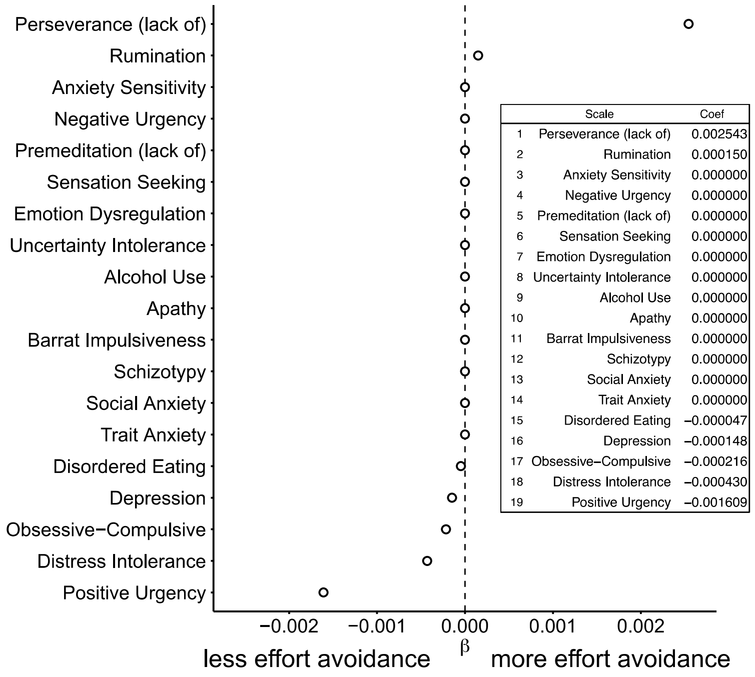

Supplemental figure S4. All self-report measures were entered into a single regression while covarying age, IQ, and gender. Elastic net regularization was applied and the inset table displays the coefficient value for each measure.

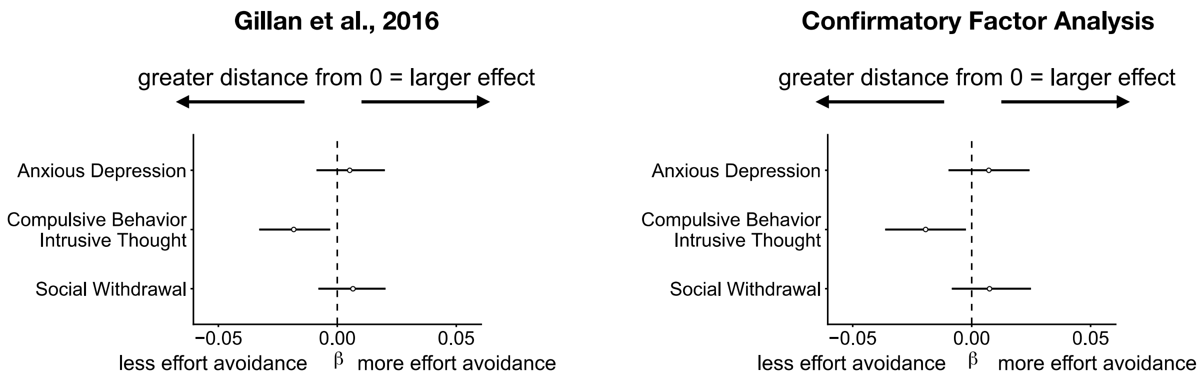

Supplemental figure S5. Comparison between regressing the Gillan (2016) loadings onto proportion low demand choices and the CFA generated loadings onto proportion low demand choices.

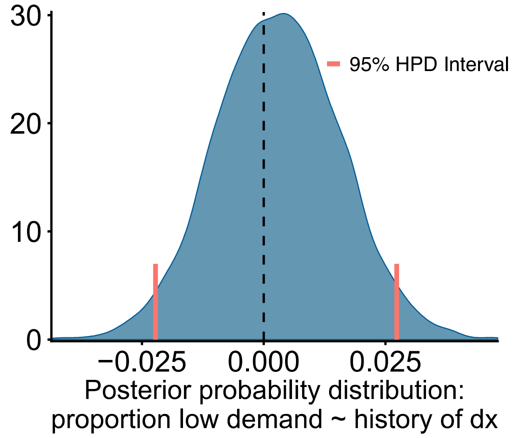

Supplemental figure S6: The posterior probability distribution when regressing history of a mental health diagnosis onto demand avoidance provided little evidence of an effect.

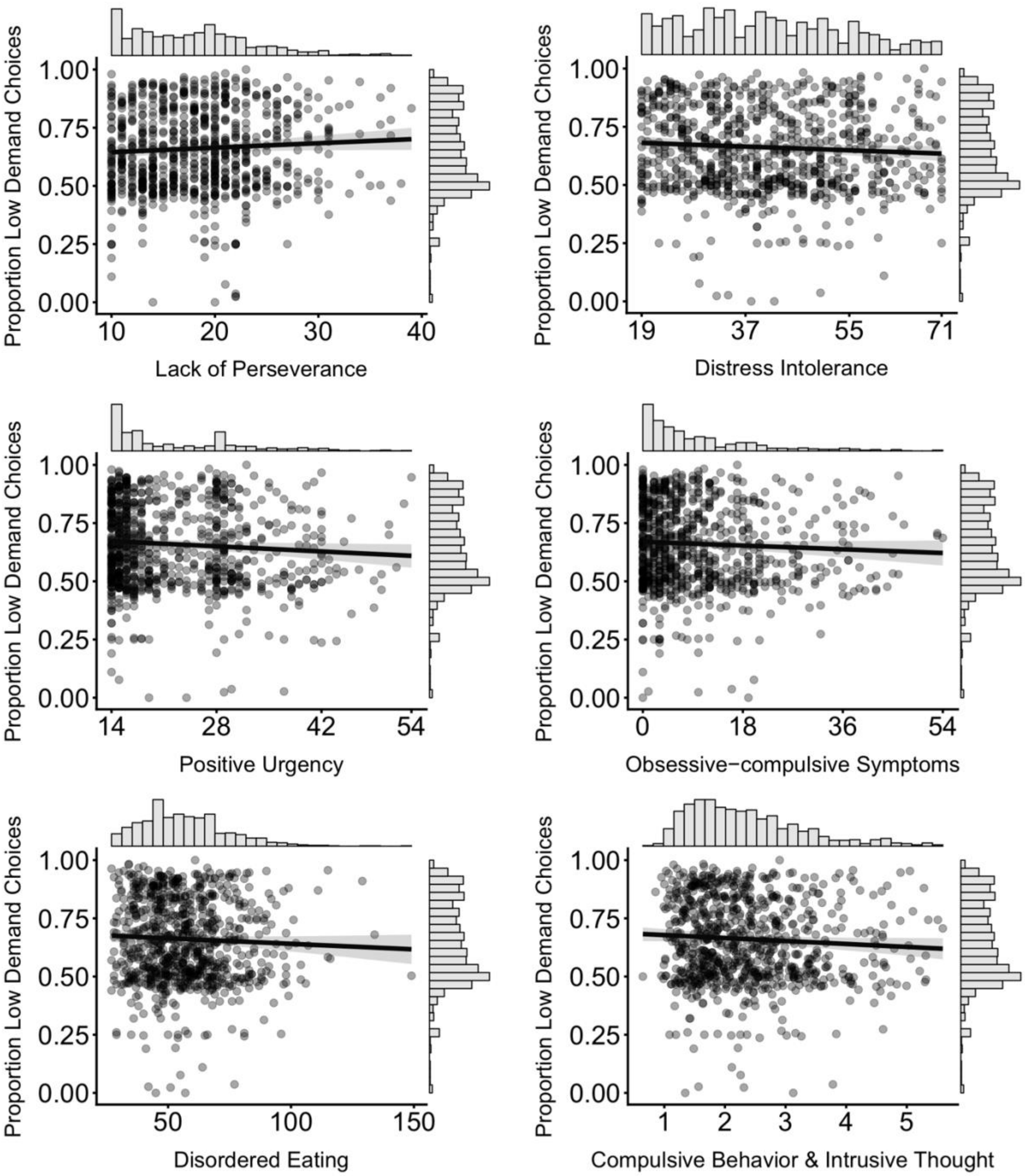

Supplemental figure S7: The constructs with the main findings are graphed with credible intervals around the regression line and marginal histograms along the axes.
